# Total Antioxidant Potential, Total Phenolic Profile and Cytotoxic Activity Against Brain Cancer: Melocan and Galdirik^§^

**DOI:** 10.17113/ftb.61.04.23.8071

**Published:** 2023-12

**Authors:** Diaa Al Yassine, Nourhane El Massri, Gunnur Demircan, Gulay Bulut, Demet Akin, Zeynep Tacer-Caba

**Affiliations:** 1 Graduate School of Engineering, Bioengineering Program, Bahçeşehir University, Istanbul, Turkey; 2 Department of Medical Biology and Genetics, Faculty of Medicine, Demiroglu Bilim University, Istanbul, Turkey; 3 Faculty of Medicine, Department of Medical Biology, Karabuk University, Karabuk, Turkey; 4 Department of Pharmacology, Bahçeşehir University School of Medicine, Istanbul, Turkey; 5 Department of Molecular Biology and Genetics, Faculty of Engineering and Natural Sciences, Bahçeşehir University, Istanbul, Turkey

**Keywords:** C6 glioblastoma, *Smilax excelsa* L., *Trachystemon orientalis*, brain cancer

## Abstract

**Research background:**

Brain cancer is known to be one of the most difficult types of cancer to cure. It has a serious impact on the lives of diagnosed people due to the insufficient treatment options and their side effects. The search for new alternative treatments is therefore ongoing. Melocan (*Smilax excelsa* L.) and galdirik (*Trachystemon orientalis*) are of great importance in both traditional culinary culture and traditional medicine around the Black Sea; however, the knowledge about their antioxidant and cytotoxic effects remains fairly limited.

**Experimental approach:**

The aim of this study is to determine the antioxidant and cytotoxic activity of *Smilax excelsa* and *Trachystemon orientalis* on the C6 glioblastoma cell line. The plants of *Smilax excelsa* and *Trachystemon orientalis* were dried and extracted and then their total phenolic content (TPC) and phenolic profiles were studied. In addition, their total antioxidant status (TAS) and total oxidant status (TOS) were determined using an assay kit. We also analysed the total antioxidant activity (TAA) using the DPPH radical scavenging assay and the cytotoxic effect on the glioma cells using the 3-(4,5-dimethylthiazol-2-yl)-2,5-diphenyltetrazolium (MTT) assay.

**Results and conclusions:**

According to the results, the water extracts of *Smilax excelsa* and *Trachystemon orientalis* had higher TPC (expressed in gallic acid equivalents on dry mass basis: 1158.17 and 262 mg/100 g, respectively) than the ethanol extracts. TAA expressed in Trolox equivalents on dry mass basis was 192.86 and 131.92 mg/100 g for *Smilax excelsa* and *Trachystemon orientalis*, respectively. The MTT assay showed that *Trachystemon orientalis* had a greater cytotoxic effect. In conclusion, the findings of the current study are promising for the development of new drugs.

**Novelty and scientific contribution:**

This is the first study that aims to evaluate the potential cytotoxic activity of two local Turkish plants, *Smilax excelsa* and *Trachystemon orientalis,* against C6 glioblastoma cells. The results confirm that both plants could be used as good therapeutic agents for the treatment of cancer in the future.

## INTRODUCTION

Cancer is a major challenge for humanity on a global scale. It is the second leading cause of death worldwide after coronary heart disorders, with a high mortality rate of 10 million deaths per year and 19.3 million new cases reported annually (*1*). Cancer is characterised by the inability to regulate or inhibit cell growth and multiplication, resulting in a tumour that can metastasize (*2*).

According to estimates from 2020, tumours that damage the brain and central nervous system were the cause of 251.329 fatalities in that year, making brain cancer the tenth deadliest disease for both sexes. Age, gender, family history (which increases the likelihood of developing brain tumour by 5 %), exposure to certain viruses and infections, head trauma, *etc*. can all have an impact on the incidence rates of brain tumour, as is the case for other types of cancer. Additionally, tumours of the central nervous system (CNS) occur in adults between the ages of 40 and 70 as well as in children. Brain cancer is a neoplasm that can grow in the brain and spinal cord (*3*). This tumour can be classified as benign or malignant based on its identification, origin and growth rate, and tumours in the latter category can metastasize to other parts of the body. In addition, there are other types of brain tumours that fall into these two main groups. One of these is glioma, a non-homogeneous group of tumours that develop from the glial cells in the central nervous system. Glioma is also considered the most common brain tumour (*4*). It has a significant impact on the quality of life because the existing treatments are ineffective. Surgical methods, radiation therapy and chemotherapeutic medications are frequently used to alleviate the pain of patients with brain cancer and increase their survival rate. However, because of the shortcomings of these therapies, there are numerous inherent limitations (*5*).

Despite the improved understanding of the fundamental causes of brain tumour development, the number of survivors with various non-benign primary brain neoplasms has not increased considerably. In fact, metastases account for the majority of deadly brain tumours. Additionally, solid tumours are common in children and are considered the primary cause of cancer-related mortality in children (*6*, *7*). All of these factors create a need for better treatment methods and researchers have recently focused on finding a therapy that is able to treat cancer with fewer adverse effects than the previously mentioned treatment methods (*8*). Natural products are now the primary focus of treatment research instead of pharmaceuticals. Plant secondary metabolites are frequently used in the medical industry. It is important to investigate these naturally occurring compounds as herbal or natural therapies in cancer treatment as they have fewer side effects, are effective, easily accessible and have the ability to overcome resistance (*9*). Many herbs used in medicine are derived from traditional medicinal plants and are used to cure a variety of diseases, including cancer (*10*). One of the main causes of cancer is the accumulation of reactive oxygen species in healthy cells. Thus, antioxidant molecules that significantly reduce the effects of oxidative stress can prevent the transformation of healthy cells into cancerous ones. The antioxidant properties of plant phytochemicals enable them to achieve this result. In addition, certain groups of polyphenolic compounds can exert other anticancer effects such as chemosensitisation, metabolic modulation, metastatic inhibition and apoptotic induction (*11*).

Melocan (*Smilax excelsa* L.) belongs to the *Smilacaceae* family, which is characterised by the woody structures and spines of its perennial members and a hight of up to fifteen meters. This plant, which is widespread in northern Anatolia, is distinguished by its spines, berry-like fruits and narrow, cylindrical toots. In spring, the plant starts producing tiny shoots that are eaten as vegetables. It is also used in cooking and it plays a significant role in many different recipes. The roots are combined and drunk as tea (*12*).

Melocan is of economic importance as it has historically been used in Anatolia to cure stomach pain and bloating; recent studies have reported its role in breast cancer treatment (*13*). The rhizomes of this plant contain phenolic and saponin compounds that are effective against oxidation, fungi and bacteria (*14*). This plant is a source of potent compounds that enable the species of this genus to fight cancer, oxidation, mutation, inflammation and bacteria (*15*). Melocan leaves have also been found to be able to protect the kidneys from CCl_4_-induced nephrotoxicity by reducing the activity of antioxidant enzymes and preventing protein and lipid oxidation reactions, thus supporting the integrity of the histological features of the kidney without restoring biological metrics. Furthermore, the plant shows similar anti-amylase and anti-glucosidase activity to acarbose, suggesting that it has the potential to treat diabetes mellitus (*12*).

Another widespread plant in eastern Bulgaria, the Caucasus and Turkey, especially along the Black Sea, is the galdirik (*Trachystemon orientalis*). This plant is considered edible since people in the Black Sea region use its flowers, rhizomes, leaves and petioles in different recipes. According to recent reports, galdirik contains anti-rheumatic, blood-purifying, diuretic, antipyretic and wound-healing properties (*16*). Additionally, large amounts of phenolic compounds, including tannin, saponin and choline have been found in galdirik in a number of studies. The effects of these compounds on antioxidants, antidiabetics, microbes and fungi have also been researched (*17*). According to Demir *et al*. (*18*), the enzyme superoxide dismutase, which is an essential component of the antioxidant defense mechanism that plants have developed to avoid or reduce the damage caused by reactive oxygen species, is present in considerable quantities in plant tissues. In addition to their antimutagenic activity (*15*), the shoots of this plant have also been found to have antimicrobial effect (*19*).

Although the different properties of these plants have been studied, their effect on glioma brain cancer cells has not yet been investigated. This aim of this study is to investigate the antioxidant effect of the water and ethanol extracts of the stems and leaves of melocan (*Smilax excelsa* L.) and galdirik (*Trachystemon orientalis*) and their cytotoxic potential against C6 glioblastoma cells.

## MATERIALS AND METHODS

### Materials

Fresh stems and leaves of *Smilax excelsa* L. and *Trachystemon orientalis* were collected in September and October 2021 in the northern Turkish village of Delikkaya near Ordu and Giresun. The settlement is 20 km from the sea at an altitude of roughly 300 m. The samples were first weeded and then washed three times to remove any remaining impurities. After being spread out thinly on a tray, they were dried in a convection oven (260644 20 GN 2/1; Electrolux Professional, Stockholm, Sweden) at 50 °C for 10 h. The samples were then stored at room temperature until use.

### Reagents

Ethanol (≥99.8 %), gallic acid (GA) standard, sodium carbonate, 2,2-diphenyl-1-picrylhydrazyl (DPPH) and 6-hydroxy-2,5,7,8-tetramethylchroman-2-carboxylic acid (Trolox) standards were from Sigma Aldrich Co., Merck (St. Louis, MO, USA), dimethyl sulfoxide (DMSO) and 3-(4,5-dimethylthiazol-2-yl)-2,5-diphenyltetrazolium bromide (MTT) were obtained from Bio Basic Inc. (Ontario, Canada) and Dulbecco's modified Eagle’s medium (DMEM) and foetal bovine serum (FBS) were procured from (Diagnovum, Ebsdorfergrund, Germany). Phosphate-buffered saline (PBS) was from WISENT Inc. (Quebec, Canada) and trypsin and sodium hydroxide were purchased from Merck KGaA (Darmstadt, Germany). Total oxidant status and total antioxidant status kits containing reagents 1 (buffer solution) and 2 (prochromogen or ABTS radical cation respectively) were obtained from Rel Assay Diagnostics (Gaziantep, Turkey).

### Proximate analyses

The total moisture content of the stems and leaves was determined using AOAC official method 925.10 (gravimetric air oven method) (*20*) and the total ash content was determined using AOAC official method 923.03 (gravimetric muffle furnace method) (*21*).

### Extraction procedures

Plant stems and leaves were used together for the analyses. Extraction methods with water and ethanol as solvents were used to prepare the plant extracts. First, the material was ground using a grinder (Scm 2934; Sinbo, Istanbul, Turkey) and then 25 mL of water were added to 5 g of the ground sample. The extract was then subjected to a series of steps, including vortexing, 15 min of sonication and 15 min of centrifugation (Rotofix 32A centrifuge; Hettich, Tuttlingen, Germany) at 8000×*g* (*22*). The same procedure was repeated to obtain a total *V*(sample extract)=100 mL for galdirik and 75 mL for melocan.

Similar procedure was used to obtain ethanol extracts. We added 25 mL of *φ*(ethanol,water)=(70 *%*) solution to approx. 5 g of sample, vortexed the mixture, sonicated for 15 min and then centrifuged at 8000x*g* for 15 min. These steps were repeated once more to obtain a total *V*(sample extract)=50 mL for both samples (*22*).

### Detection of the total phenolic content and major phenolic compounds

The total phenolic contents of the samples were assessed using the Folin-Ciocalteu colourimetric technique (*22*). First, 100 mL of plant extract and 750 mL of 6 % sodium carbonate solution were added to 750 mL of a Folin-Ciocalteu reagent solution diluted ten times with distilled water. After 1.5 h of incubation in the dark, the tubes were vortexed and absorbance was measured at 750 nm using a Nanodrop spectrophotometer and plate reader Multiskan GO (Thermo Fischer Scientific, Dreieich, Germany). The mass fraction of phenolic compounds was determined using the gallic acid solution standard curve and is reported in mg of gallic acid equivalents per 100 g of dry mass. The analyses were carried out in triplicate.

High-performance liquid chromatography (W600 HPLC system with a photodiode array (PDA) detector; Waters Turkey, Istanbul, Turkey) was used to determine the main phenolic compounds in the plant samples. A Luna C18 column (150 mm×4.60 mm pore size, 100 Å, particle size 5 µm; Phenomenex, Torrance, CA, USA) was used as the stationary phase, while solvent A (Milli-Q water with trifluoroacetic acid *φ*(TFA)=0.1 %) and solvent B (acetonitrile with *φ*(TFA)=0.1 %) were used as the mobile phase (*23*). External standard calibration curves were used for quantification. All samples and calibration solutions were filtered through a 0.45-µm membrane filter and 2 mL of the filtered sample were placed into vials. The flow rate was 1 mL/min. Detections were performed at wavelengths of 280, 312 and 360 nm.

### Determination of total antioxidant activity

The total antioxidant activity of the galdirik and melocan extracts was determined by their DPPH radical scavenging abilities (*24*). To achieve this, the plant extracts of this mixture were added to 2 mL of a freshly made solution that contained 1 mmol/L of DPPH reagent. After 30 min in the dark, the test tubes were brought back into the light to measure the absorbance at 517 nm. The results of this experiment are presented on dry mass basis as mg Trolox equivalent (TE) per 100 g of the plant material.

### Total antioxidant status and total oxidant status

The total antioxidant status (TAS) of both extracts was evaluated using commercial TAS assay kit (Rel Assay Diagnostics). This test shows how hydrogen peroxide oxidises the free radical ABTS (2,2'-azino-bis(3-ethylbenzothiazoline-6-sulfonic acid)), which causes ABTS to lose its dark blue colour and become stable. The higher content of antioxidants, the more the colour intensity decreases. To achieve this, 18 µL of water and ethanol sample extracts, standard, or distilled water were combined with 300 µL of reagent 1. After 30 s, the absorbance (*A*_1_) was measured spectrophotometrically (Nanodrop spectrophotometer and plate reader Multiskan GO, Thermo Fischer Scientific) at 660 nm. The mixture was then mixed with 45 µL of reagent 2 and the absorbance (*A*_2_) was measured five minutes later at 37 °C and 660 nm. The total antioxidant status (TAS) was determined using the following formula:


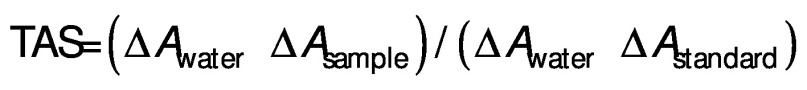
 /1/

where


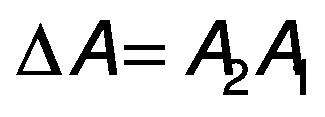
 /2/

Additionally, the oxidant status of both extracts was assessed using total oxidant status (TOS) commercial assay kit (Rel Assay Diagnostics). This assessment is based on the oxidation of Fe^2+^ to Fe^3+^ which reacts with xylenol orange to form a purple complex that can be measured spectrophotometrically. In this test, 45 µL of sample or standard and 300 µL of reagent 1 were combined and the absorbance (*A*_1_) was measured spectrophotometrically at 530 nm after 30 s. A volume of 15 µL of reagent 2 was added to the mixture and after incubation at 37 °C for 5 min, the absorbance (*A*_2_) was measured. The TOS was calculated using the following formula:


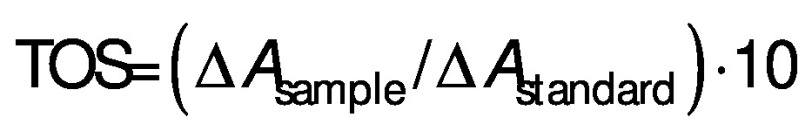
 /3/

where Δ*A* is calculated as in Eq. 2 and 10 is the concentration of standard solution of H_2_O_2_ in μmol/L.

### Cytotoxicity

#### C6 glioblastoma culture

C6 glioblastoma cells were obtained from the American Type Culture Collection (ATCC, Rockville, MD, USA). The cells were maintained in DMEM with 10 % heat-inactivated FBS and 1 % penicillin/streptomycin solution as supplements and the cytotoxic effect of water and ethanol extracts of melocan and galdirik on them was evaluated.

#### Cell culture passaging protocol

For the trypsinization of the cells, the cell culture medium was discarded and the cells were washed with PBS. Trypsin (1 mL) was added to the cells and then the flask was placed in the CO_2_ incubator (EC 160; NÜVE, Ankara, Turkey) for 3 min (5 % CO_2_, 37 °C). The flask was then removed from the incubator, examined under an inverted microscope (CK40; Olympus, Tokyo, Japan) for the presence of single cell suspensions, and tapped twice to ensure that every cell had detached completely from the flask surface. A volume of 1 mL of DMEM was then added to terminate the trypsinization. The cells were resuspended by pipetting and transferred to a 15-mL centrifuge tube (ISOLAB, İstanbul, Turkey) where they were centrifuged for 2 min at 2000×*g* in an Eppendorf 5810 R centrifuge (Eppendorf, Hamburg, Germany). After the aspiration of the supernatant, 2 mL of DMEM were added to the pellet. This volume was divided into two Falcon tubes, to which 9 mL of DMEM were added to bring the total volume to 10 mL.

The cytotoxic effect of melocan and galdirik water and ethanol extracts on human glioma cancer cells was investigated over a period of 24 h using the MTT assay. The cells were incubated for 72 h at a density of 5·10^3^ cells/mL in a 96-well flat-bottomed cell culture plate. After removing the medium, the cells were treated with different concentrations of plant extracts (ranging from 80 to 5 µg/mL) for 48 h. The experiment was carried out in triplicate, using three wells for each concentration. To dissolve the crystals, DMSO was added to each well after the addition of 10 µL MTT solution. Finally, the absorbance (*A*) was measured at 570 nm (Nanodrop spectrophotometer and plate reader Multiskan GO (Thermo Fischer Scientific). The obtained values were used to calculate the percentage of cell viability using the following equation:


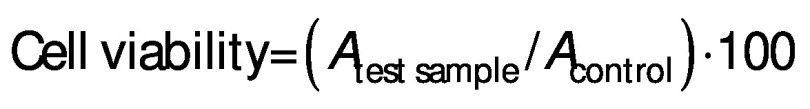
 /4/

The logarithmic graph of the log concentration *versus* the percentage of cell viability was used to calculate the IC_50_. The IC_50_ value is the concentration (in µg/mL) that inhibits cell growth by 50 % (*19*).

### Statistical analysis

All results were from at least two repetitions of experiments and the results are presented as mean value±standard deviation. The data were analysed using the IBM SPSS Statistics software v. 20 (*25*). A paired t-test was used to analyse the differences between the two extracts (water and *φ*(ethanol, water)=70 %) of each plant to determine whether they were statistically significant (p<0.05) (*26*). Analysis of variance (ANOVA) was used for the TAS/TOS and cytotoxic activity tests and the Duncan test was the *post-hoc* evaluation method used to determine the differences. Differences between samples were calculated at 95 % significance level. Pearson’s correlation matrix was used to detect the correlations between the antioxidant activity and the total phenolic contents of the samples (p<0.01 and p<0.05) were calculated using the same software.

## RESULTS AND DISCUSSION

### Composition of raw materials

The total moisture and ash mass fractions of melocan and galdirik samples were determined and shown in Table 1.

**Table 1:** Proximate components, total phenolics and antioxidant activity of the samples

Sample	*w*(moisture)/%	*w*(ash)/%	*w*(total phenolics as GAE)/(mg/100 g)	*w*(TAA as TE)/(mg/100 g)
Melocan	12.36±0.05	8.5±0.2	nd	nd
Melocan water extract	nd	nd	(1158±4)^a^	(192.8±5.5)^a^
Melocan 70 % ethanol extract	nd	nd	(293.89±0.01)^b^	(154.7±0.8)^a^
Galdirik	15.92±0.8	29.0±0.2	nd	nd
Galdirik water extract	nd	nd	(262.6±1.6)^a^	(132.6±0.1)^a^
Galdirik 70 % ethanol extract	nd	nd	(235.7±8.8)^a^	(73.4±8.5)^a^

Values are presented as mean±standard deviation of duplicate analyses. Values of each sample in the same column with different letters (a-b) differ significantly (p<0.05). GAE=gallic acid equivalent, TAA=total antioxidant activity, TE=Trolox equivalent, nd=not determined

The moisture mass fraction of the dried leaves and stems of melocan was 12.36 % due to the drying step, while the moisture mass fraction of the fresh edible parts of the plant was 82.6 % in a previous study (*27*). The ash content of melocan (8.5 %) determined in this study is consistent with previous findings in the literature, where the ash content was 6.77 % (*28*) and 7.10 % (*29*).

The results for galdirik (29.0 %) were slightly higher than the values reported in the literature; the ash mass fraction of the different genotypes of *T. orientalis* was between 9.2 and 17 % in a previous study (*30*). This could be related to the use of the whole plant in the study.

### Total phenolic content and main phenolic compounds detected in plant extracts

The total phenolic content of the water and 70 % ethanol plant extracts was determined (see Table 1), as the ability of plant phenols to scavenge free radicals is one of the most important anticancer mechanisms.

The total phenolic contents on dry mass basis, expressed as GAE, in the melocan stems and leaves were 1158 and 293.89 mg/100 g (Table 1) for the water and 70 % ethanol extracts, respectively, indicating a significantly higher (p=0.003) mass fraction of phenolic compounds in the water extract. These results are consistent with other findings in the literature, which show that the water extracts of the leaves of these plants contain higher mass fractions of phenolic compounds than the ethanol and infusion extracts (*31*). However, other researchers (*12*) have reported that the total phenolic content on dry mass basis of the hexane extract of the leaves was 1930 mg/100 g and the hexane extract of the stems was 710 mg/g, showing that the leaves of this plant contain higher mass fraction of phenolic compounds than the stems. On the other hand, in the present study, the combination of these two plant parts resulted in a favourable amount of phenolic compounds. In contrast to these results, another research group found that the phenolic content on dry mass basis of the leaves of melocan was 3060 and 3010 mg/100 g in water and ethanol, respectively, which was higher than in this study (*31*). The differences in the methodology and plant parts used could be the reason for these differences.

The total phenolic content on dry mass basis, expressed as GAE, of the galdirik water and 70 % ethanol extracts was 262.6 and 235.7 mg/100 g, respectively. The difference is statistically insignificant (p=0.165). In previous studies on galdirik, the total phenolic content was 9000 mg/100 g in water extracts and 2120 mg/100 g in ethanol extracts (*32*). The values found in the present study are lower than those found in the literature. They also show that the galdirik water extract has a significantly higher total phenolic content. These discrepancies can be attributed to the different extraction techniques, extraction solvents, initial concentrations, and the effects of each plant part used (*16*).

**Fig. 1:**
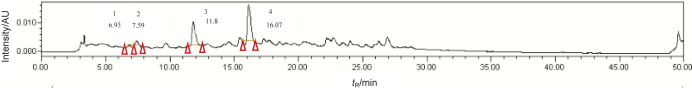
HPLC chromatogram of melocan at *λ*=312 nm. Phenolic compounds in the sample are coded with numbers: 1=protocatechuic acid, 2=unidentified compound, 3=chlorogenic acid and 4=chlorogenic acid derivatives. *t*_R_=retention time

The main phenolic acids found in melocan were (in mg/100 g): protocatechuic acid (5.35±0.04), chlorogenic acid (5.91±0.02) and chlorogenic acid derivatives (9.76±0.03). The HPLC chromatogram of melocan is given in Fig. 1. Melocan has been reported in the literature to contain lanesol, violasterol A, *trans*-resveratrol, 5-O-caffeoylshikimic acid and 6-O-caffeoyl-β-d-fructofuranosyl-(2-1)-α-d-glucopyranoside (*33*). In another study, the researchers reported the following phenolic components in melocan shoot samples (in mg/100 g): caffeic acid 0.46, ferulic acid 9.38, rosmarinic acid 0.03 and hydrocynnamic acid 0.03, noting that a comparison with the literature is not possible (*34*). Clear peaks were not determined in galdirik. This could be related to the properties of the extract or the HPLC measurement conditions. Only rutin and myricetin were detected in abundance in infusions of galdirik leaves and stems (*35*) and in another paper gallic, vanillic and rosmarinic acids were reported in galdirik (*36*).

### Antioxidant potential of plant extracts

Assessing the radical scavenging activity of plants is a fundamental step in determining their anticancer activity since oxidative stress is one of the main causes of cancer. The total antioxidant activity (TAA) of melocan and galdirik extracts was measured using the DPPH radical scavenging activity method and the results are shown in Table 1. The DPPH radical scavenging method determines the quantity of DPPH radicals reduced by antioxidants that provide hydrogen or transfer electrons to generate DPPH-H, a non-radical version of the DPPH radical.

In this study, the antioxidant content of melocan was found to be higher in the water extract (192.8 mg/100 g) than in the 70 % ethanol extract (154.7 mg/100 g) (p>0.05) (Table 1).

According to a previous study (*37*), melocan has a strong antioxidant activity of 62.36 mmol/kg measured with ferric reducing ability of plasma (FRAP) method. This result is comparable to another study that used the β-carotene bleaching method and the linoleic acid system and concluded that this plant has a significant antioxidant effect. In addition, using the phosphomolybdenum method, this plant was found to have a high antioxidant content measured as α-tocopherol (almost 1200 µg/g).

Our findings suggest that the phenolic compounds in melocan contribute significantly to its antioxidant activity because there is a positive and linear correlation between the total phenolic content and the total antioxidant content.

The total antioxidant activity, expressed in Trolox equivalents, of galdirik extracts was 132.6 and 73.4 mg/100 g of the water and 70 % ethanol extracts, respectively (Table 1). As with melocan, the water extract of galdirik had a higher antioxidant activity than the 70 % ethanol extract (p=0.165). The antioxidant activity of the water and ethanol extracts of galdirik was measured in a previous study (*32*) using the ABTS free radical scavenging activity assay, and the results were 1725 and 240 mmol/kg, respectively. In that study, the antioxidant concentration of this plant was comparable to that of broccoli and asparagus extracts, which had a value of 26.2 mmol/kg.

According to the results of the correlation assessment, total phenolic content and total antioxidant activity correlated very well (p<0.01) with both the water and 70 % ethanol extracts (0.998 for the 70 % ethanol extracts and 0.993 for the water extracts; data not shown).

### TAS/TOS of plant extracts

**Fig. 2:**
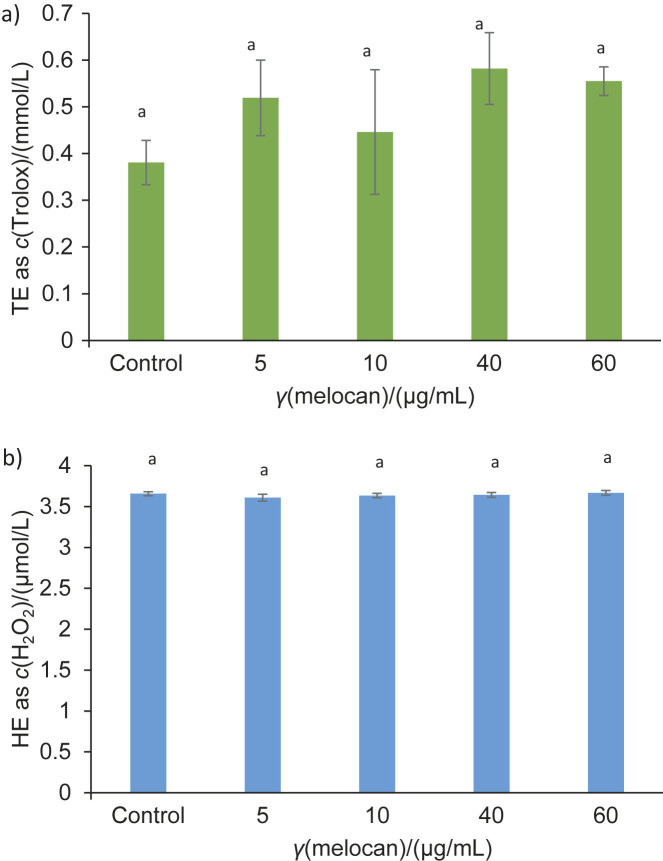
Results of melocan as mean value±standard deviation for: a) total antioxidant status (TAS) and b) total oxidant status (TOS). There were no significant differences between the samples in the same bar (p<0.05)

**Fig. 3:**
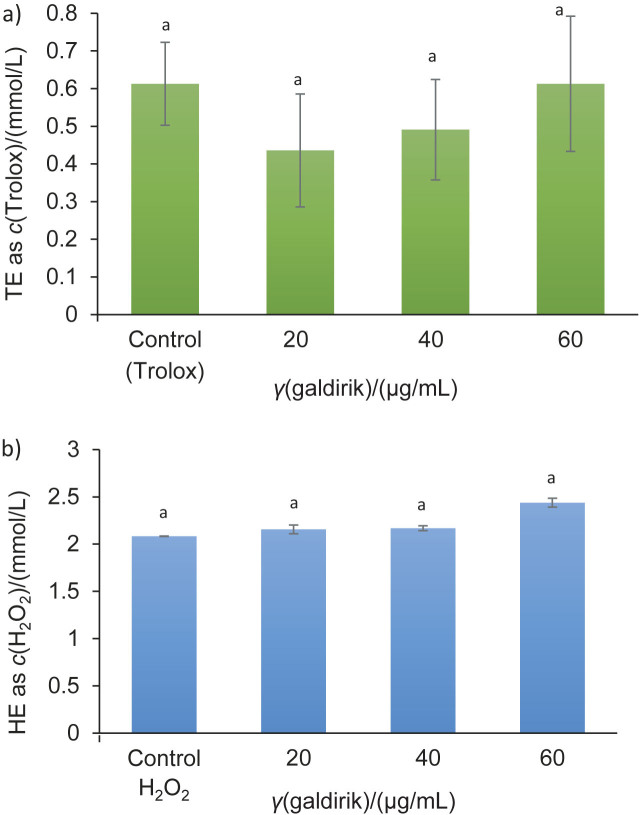
Results of galdirik as mean value±standard deviation for: a) total antioxidant status (TAS) and b) total oxidant status (TOS). There were no significant differences between the samples in the same bar (p<0.05)

The TAS value is an indicator that shows the activity of antioxidant compounds, while the TOS value shows the oxidant compounds produced by sample extracts. In the presence of the C6 glioblastoma cell line, the TAS and TOS were calculated for different amounts of melocan and galdirik water extracts, as shown in Fig. 2 and Fig. 3, respectively.

The TAS and TOS values of melocan have never been the subject of a previous study. In the current study, the water extract of a combination of stems and leaves had an average TAS value (as Trolox equivalent) of 0.6 mmol/L (Fig. 2a) and a TOS value (as H_2_O_2_ equivalent) of 3.64 μmol/L (Fig. 2b). Compared to the control, the TAS value of the melocan water extract was higher, although not statistically significant (p=0.416). According to the results, melocan does not exhibit significant oxidant activity, as the TOS value remains almost the same as that of the control sample. The antioxidants and oxidants that melocan produces as a result of environmental or metabolic processes are represented by the TAS and TOS values and no significant oxidants in samples were detected (changing between 0.38 and 0.58 mmol/mL and between 3.61 and 3.67 μmol/L for TAS and TOS, respectively).

The TAS value (as TE) of galdirik is on average 0.5 mmol/L (Fig. 3a), while the TOS value (as H_2_O_2_) is 2.3 μmol/L (Fig. 3b). The results of the study show that the TAS value increases with the increasing concentration of the water extract and becomes comparable to the control at a concentration of 60 µg/mL (p>0.05). The TOS for the same concentration of galdirik extract (60 µg/mL) had a value of 2.4 μmol/L, which is slightly higher (p>0.05) than that of the control (Fig. 3b).

### Cytotoxic activity of plant extracts determined by MTT assay

**Fig. 4:**
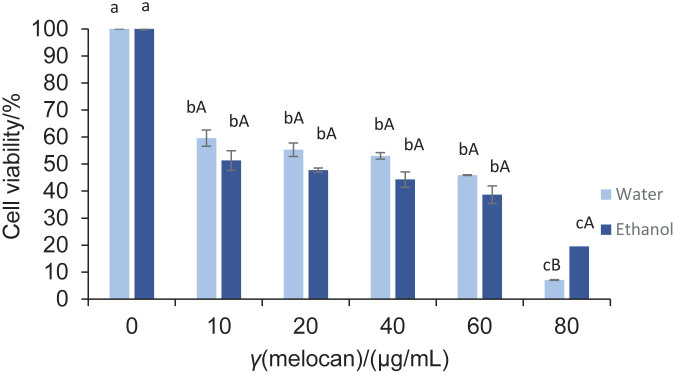
The cytotoxic activity of water and 70 % ethanol extracts of melocan. Results are presented as mean value±standard deviation of cyototoxic activity, *N*=2. Different lower-case letters among the concentrations of an extract in water or 70 % ethanol indicate significant difference (p<0.05) and different capital letters at each concentration of different extracts indicate significant difference (p<0.05)

**Fig. 5:**
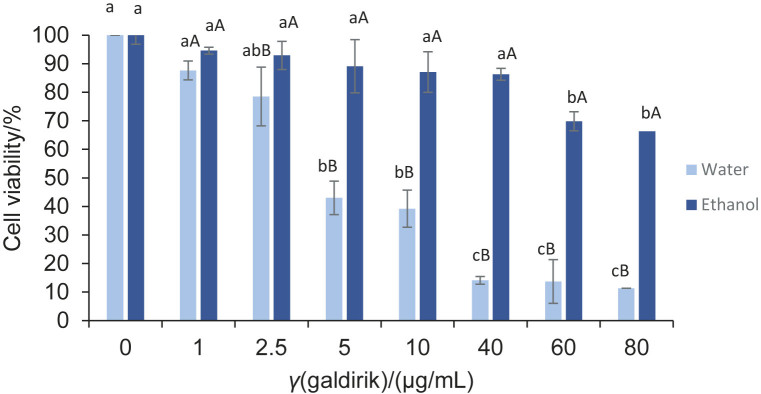
The cytotoxic activity of water and 70 % ethanol extracts of galdirik. Results are presented as mean value±standard deviation of cyototoxic activity, *N*=2. Different lower-case letters among the concentrations of an extract in water or 70 % ethanol indicate significant difference (p<0.05) and different capital letters at each concentration of different extracts indicate significant difference (p<0.05)

The cytotoxic activity of the water and 70 % ethanol extracts of melocan and galdirik on the C6 glioblastoma cancer cells was examined using the MTT assay and the results are shown in Fig. 4 and Fig. 5, respectively. MTT is reduced at the ubiquinone and cytochrome b and c sites of the mitochondrial electron transport system. MTT is a colourimetric assay often used to determine the cell proliferation, viability and cytotoxicity. It shows cytotoxicity of test sample. Essentially, when a cell is alive, its metabolism causes the yellow tetrazolium dye to be reduced into purple formazan crystals, which are subsequently dissolved with the addition of DMSO (*38*). Thus, this method is used to quantify the colour change as a means of determining the number of cells that survived in the final stage.

According to the results of the current study, the melocan extracts exerted a cytotoxic effect on the C6 glioblastoma cell line, with a higher effect of the water extract (IC_50_=7.73 µg/mL) than of the 70 % ethanol extract (IC_50_=10.05 µg/mL) (p>0.05) (Fig. 4). These results are in agreement with the results of total phenolic and antioxidant activity, which also showed that the water extract of this plant is more effective than the 70 % ethanol extract.

However, in a previous study, several components extracted from melocan were found to have a cytotoxic effect on the MCF-7 cell line (*33*). According to the same study, the violasterol A and solanesol had the greatest inhibitory effects on these types of breast cancer cells, with IC_50_ values of 190.0 and 161.6 μM, respectively. In addition, *Smilax* genus was found to be unique due to its large amounts of steroidal saponins, compounds that fall into the category of secondary metabolites and contribute to the biological activity of many medicinal plants, and particularly to their cytotoxic effects (*39*). Apoptosis and cytotoxicity in human osteosarcoma (U2OS) cells have been demonstrated in *Smilax aspera*, one of the species of this genus (*40*). The same plant was associated with cytotoxic effect on lung cancer cells (*41*) as well as on ovarian adenocarcinoma (OVCAR3), lung carcinoma (A549) and breast cancer (MDA-MB-231) cell lines (*42*).

The present results show that the water and 70 % ethanol extracts of galdirik were able to inhibit cell viability and proliferation of C6 glioma cells in a dose-dependent manner (Fig. 5). The cell viability of the water extract was 87.65 % at 1 µg/mL and decreased as the concentration increased, reaching 11.33 % at 80 µg/mL. Similar to water extract, the 70 % ethanol extract also had a cytotoxic effect on the C6 cells, but less effective. In water extracts, cell viability peaked at 94.59 % at 1 µg/mL and dropped to 66.34 % at 80 µg/mL. Additionally, the IC_50_ value for water extract was 4.47 µg/mL, meaning that it was more efficient than the ethanol extract as 50 % inhibition of cell viability was not achieved even at 80 µg/mL. The IC_50_ value indicates how much drug is needed to inhibit a biological process by half, and lower IC_50_ value indicates higher effect. Therefore, the results presented here show that the water extract is more cytotoxic than the ethanol extract, supporting the earlier finding of this study, *i.e*. that the water extract has a more pronounced antioxidant activity due to its higher total phenolic content. For comparison, it has been documented in the literature (*43*) that an ethanol extract (70 %) from a different plant, *Rhododendron brachycarpu*, has an anticancer effect on a number of human cancer cell lines, including the MCF-7 breast cancer cell line, A549 lung cancer cell line and Hep3B liver cancer cell line, among others. However, to accurately determine the cytotoxic activity and exact mechanism of action of these plants on glioblastoma, it is necessary to investigate the blood-brain barrier crossing properties of their specific phenolic compounds (*e.g*. from flavonoids) (*44*). Therefore, more extensive studies are needed.

## CONCLUSIONS

Overall, the results of this study show for the first time that both plants, galdirik and melocan, have the potential to be used in the treatment of glioblastoma cancer. Our results are consistent with the literature data and highlight *de novo* the very important role of natural products in cancer research, where both plants could be unique and good therapeutic agents for cancer therapy in medical care. Specifically, the galdirik water extract has a lower IC_50_ value. This study can serve as a guide for future research that aims to identify the exact pathways controlled by this plant that have an effect on glioblastoma multiform. To confirm the *in vivo* effects, the molecular mechanism(s) of the cytotoxic activity of the plant need to be specified and certain specialised components of the plants need to be investigated.
